# Evaluation of the Benefit of Routine Intraoperative Frozen Section Analysis of Sentinel Lymph Nodes in Breast Cancer

**DOI:** 10.1155/2013/843793

**Published:** 2013-09-16

**Authors:** C. M. T. P. Francissen, R. F. D. van la Parra, A. H. Mulder, A. M. Bosch, W. K. de Roos

**Affiliations:** ^1^Department of Surgery, Gelderse Vallei Hospital, 6716 RP Ede, The Netherlands; ^2^Department of Pathology, Rijnstate Hospital, 6518 AD Arnhem, The Netherlands

## Abstract

*Aims*. Intraoperative analysis of the sentinel lymph node (SLN) by frozen section (FS) allows for immediate axillary lymph node dissection (ALND) in case of metastatic disease in patients with breast cancer. The aim of this study is to evaluate the benefit of intraoperative FS, with regard to false negative rate (FNR) and influence on operation time. *Materials and Methods*. Intraoperative analysis of the SLN by FS was performed on 628 patients between January 2005 and October 2009. Patients were retrospectively studied. *Results*. FS accurately predicted axillary status in 525 patients (83.6%). There were 78 true positive findings (12.4%), of which there are 66 macrometastases (84.6%), 2 false positive findings (0.3%), and 101 false negative findings (16.1%), of which there are 65 micrometastases and isolated tumour cells (64.4%) resulting in an FNR of 56.4%. Additional operation time of a secondary ALND after wide local excision and SLNB is 17 minutes, in case of ablative surgery 35 minutes. The SLN was negative in 449 patients (71.5%), making their scheduled operation time unnecessary. *Conclusions*. FS was associated with a high false negative rate (FNR) in our population, and the use of telepathology caused an increase in this rate. Only 12.4% of the patients benefited from intraoperative FS, as secondary ALND could be avoided, so FS may be indicated for a selected group of patients.

## 1. Introduction

Axillary lymph node status is still considered the most important prognostic factor in patients with breast cancer. With the ongoing improvement of breast cancer screening programs, more patients are diagnosed at an earlier stage, leading to less nodal involvement. Sentinel lymph node biopsy (SLNB) has been established as a reliable method to evaluate the lymph node status of the axilla, making standard axillary lymph node dissection (ALND) unnecessary. Compared to ALND, SLNB is associated with less morbidity. Intraoperative analysis of the sentinel node by frozen section (FS) allows for immediate ALND when a metastasis is found in the sentinel node, thus avoiding a second procedure. However, among the drawbacks of FS are (1) the possibility of false negative and false positive results and (2) increase in operation time, because extra time is scheduled in advance in case the FS turns out to be positive. The sensitivity of FS has been reported to range from 58% to 76%, depending on tumour characteristics (e.g., tumour size) and the method of pathological examination [1–6]. 

This study was designed to evaluate the benefit of FS in our hospital, with regard to the false negative rate (FNR) and true positive results, as well as the additional operation times. By comparing the operation times of the different procedures we evaluated if intraoperative FS of the SLNB is either time saving or time consuming. 

## 2. Materials and Methods

### 2.1. Patients

From a prospectively collected database of breast cancer patients, 628 patients with invasive breast cancer who underwent SLNB with FS between January 1st, 2005 and October 1st, 2009 were selected. Patients with ductal carcinoma in situ and/or failure of the SLNB procedure were excluded. None of the selected patients were treated neoadjuvant before SLNB. Operation times were retrospectively collected from a separate database, which is kept by the department of anaesthesiology. 

### 2.2. Lymph Node Mapping

Sentinel lymph node (SLN) mapping was performed by using lymphoscintigraphy with or without patent blue dye. On the day of operation, lymphoscintigraphy was performed by injecting 77 MBq 99-Tc nanocolloid in different depots (total amount 0.8 mL) peritumoural or periareolar. Scans of the involved breast and axilla were first acquired 30 minutes after injection. In case of no visible activity, scans were repeated 2 hours after tracer injection. Patent blue dye was injected immediately before surgery. During surgery, the SLN was localized by using a gamma probe. All blue and/or radioactive nodes counting 10-fold ex vivo relative to the background were regarded as SLNs and sent for FS. 

### 2.3. Pathological Examination

SLNs with a diameter of more than 5 mm were bisected longitudinally and frozen. SLNs with a diameter of less than 5 mm were frozen intact. Frozen sections were taken with a microtome setting of 4 *μ*m. Until January 1st, 2007, a pathologist was present in our hospital for FS analysis, after this date, telepathology was used. A digital image of the FS was made and sent to the pathologist who examined this image via a screen. During the period of telepathology, there was also a switch in equipment. The remaining nodal tissue was fixated in 10% formalin and embedded in paraffin. After this fixation serial sections were made of the SLN for definitive analysis. Macrometastases were defined as a diameter > 2 mm, micrometastases as a diameter between 0.2 and 2 mm, and isolated tumour cells (ITCs) as single tumour cells or small clusters of cells (diameter < 0.2 mm). 

### 2.4. Statistical Analyses

Statistical analyses were performed by using SPSS 15.0. Diagnostic performance was described in sensitivity and false negative rate (FNR) and *P* values were calculated for sensitivity and FNR.

## 3. Results

FS of the SLN was performed on 628 patients (of which 2 were males). The mean age was 60.3 years (median 59.9 years, range 30–88 years). Most of the patients were diagnosed with a ductal carcinoma (472 patients; 75.2%) or lobular carcinoma (80 patients; 12.7%). In 399 patients (63.4%), wide local excision (WLE) was performed; the remaining 229 patients underwent mastectomy. The mean tumour size was 1.62 cm (median 1.5 cm, range 1–6.3 cm).

FS accurately diagnosed the status of the SLN in 83.6% of the patients (Table 1). FS was negative in 548 cases (87.3%) and positive in 80 cases (12.7%). When FS displayed a metastasis, ALND was performed in the same procedure. Definitive pathology revealed a negative sentinel node in 449 cases (71.5%) and metastatic disease in 179 cases (27.6%). FS was false negative in 101 cases (16.1%) and false positive in 2 cases (0.3%). In the group of patients with a false negative FS, 69 patients (68.3%) underwent subsequent ALND; the remaining 32 patients received adjuvant radiation therapy of the axilla or adjuvant systemic therapy. Decisions on alternative treatment were based on multidisciplinary breast cancer team consultation and based on individual discussions between patient and surgeon. Of all 628 patients who underwent intraoperative FS, 78 patients (12.4%) benefited from this procedure as immediate ALND was performed. The sensitivity of FS, defined as (true positive)/(true positive + false negative), was 43.6%. When separated by T-status, sensitivity was 41.4% for T1-tumours and 47.1% for T2/T3 tumours (*P* value 0.456, Table 1). False negative rate (FNR), defined as (false negative)/(true positive + false negative), was 56.4%. Separated by T-status, FNR was, respectively, 58.6% for T1 and 52.9% for T2/T3.

In the group of patients with a positive SLNB, the percentages of ITCs, micro- and macrometastases were 25 (14.0%), 52 (29.1%), and 101 (56.4%), respectively. In one patient, this specific information was missing. FS was less sensitive for detecting micrometastases and ITCs (sensitivity 21.1% and 4.0%, resp.) than for detecting macrometastases (sensitivity 65.3%) (*P* value macro- versus micrometastases 0.001). Micrometastases and ITCs are more often seen in T1 staged tumours (47.7% versus 38.2%, Table 2).

Results were also analysed by the changes in FS examination techniques during the study period (Table 3). Since the introduction of telepathology, the sensitivity and FNR worsened. A further deterioration is seen after the change in equipment. When compared to FS examination by a pathologist at site, the results of this second period of telepathology were significantly worse (*P* = 0.025).

### 3.1. Operation Times

**Figure 1:**
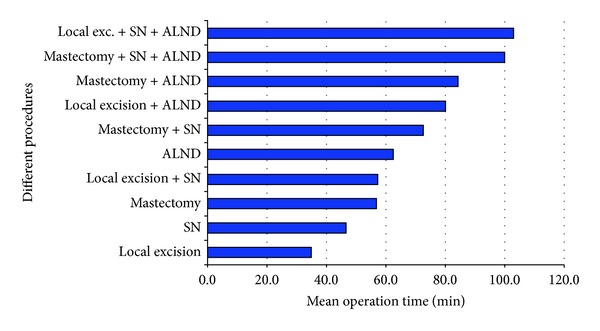
Different operation times of separate and combined procedures. SN: sentinel node, ALND: axillary lymph node dissection, and exc: excision.

Operation time was reported as the time from incision to closure. Wide local excision (WLE) combined with a SLNB and a negative FS has a mean operation time of 57.3 minutes (range 25–162). If FS was positive and ALND was performed immediately, the mean operation time was 102.7 minutes (range 52–178). A mastectomy with SLNB takes 72.6 minutes (range 23–140); when completed with an ALND, the mean operation time was 100.2 minutes (range 51–178). In comparison, an ALND as a separate procedure takes more than one hour. Operation times of all different procedures in breast surgery are shown in Figure 1.

## 4. Discussion

Intraoperative analysis of the SLN by FS allows for immediate ALND in case of metastatic disease. In this population of 628 patients who underwent SLNB, 78 patients (12.4%) did benefit from intraoperative FS examination. In 101 cases FS was false negative. These patients had to undergo ALND in a separate procedure. Sensitivity was 43.6% overall; when divided by T-status, sensitivity varies from 41.4% for T1-tumours to 47.8% for T2-tumours. There were few T3-tumours in this population, making this number unreliable. Weiser et al. [2] demonstrated that sensitivity is dependent on tumour size. They also demonstrated a higher sensitivity of FS when the SLNB contained macrometastases compared to micrometastases or ITCs (65.3% versus 21.1% or 11.1%). A similar correlation between tumour and metastasis size was also seen in our population. Patients with T2/T3 tumours had relatively more macrometastases in the FS.  Table 4 presents an overview of recent studies on sensitivity and false negative rate of FS in the literature. The sensitivity in these studies ranges from 55.6% to 83.6%; the false negative rates range from 16.3% to 44.4%. 

Despite high volumes of breast surgery, our clinic does not have an in-hospital pathology department, and therefore telepathology was introduced in 2007. The difference in outcome of sensitivity and FNR can be partially explained by this introduction. The further deterioration seen after the change in equipment is hard to explain. Sensitivity decreased from 52.3% to 32.0%. There were few T3 tumours and macrometastases in our population. However, some authors (Weiser et al., van de Vrande et al., and Wada et al.) also had few T3-tumours in their population and still had a lower FNR [2, 3, 7]. Due to the correlation between tumour diameter and positive intraoperative examination of the sentinel node, Fortunato et al. suggested that FS is particularly helpful in T2 patients [4]. Finally, the very low sensitivity and high false negative rates for intraoperative FS of sentinel nodes are worse than what the literature suggests, may be due to publication bias. However, this may also be due to an increasing recognition of micrometastases.

We also evaluated the benefits of FS by comparing operation times. The difference in operation time is 17.2 (in case of WLE) and 35 minutes (in case of mastectomy) in favour of ALND in the same procedure, so SLNB does not seem to be time consuming. However, this applies to the patients with a positive sentinel node. In this study, 28.5% of the SLNBs were positive, which means that 71.5% had a negative sentinel node. For these patients, extra operation time was scheduled because of the possibility of a positive FS and thus subsequent ALND. This means that the FS procedure causes an element of uncertainty in the operation room schedule and consumed unnecessary scheduled operation time because additional operation time is scheduled in every patient undergoing breast surgery with SLNB. Patients with a true positive FS benefit from this procedure, because they undergo immediate ALND, thereby avoiding a second operation. Disadvantages of a second procedure are the increased costs, the potential additional morbidity, and the negative emotional impact on the patient [11–13].

Rónká et al. showed that, with respect to hospital costs, FS analysis seems to be worthwhile as long as the false negative rate does not exceed 35% [14]. This is due to the lower costs of a shorter hospital stay and the association with a decrease in long-term postoperative morbidity. Holm et al. performed a cost analysis and concluded that intraoperative examination of the SNB by immunohistochemical staining gave an overall cost saving: the cost saved by avoiding reoperations exceeded the added cost of examination [15]. The cost/benefit balance of FS examination is still being debated, and Zavagno et al. opted to reserve intraoperative histology only for patients with larger tumours, who have a higher risk of nodal metastases [16]. In selecting those women, a nomogram can be useful as a decision aid [17]. Goyal et al. compared delayed ALND with immediate ALND and observed an increased axillary operation time and total hospital stay in case of the two-step procedure [18]. It is still unclear whether intraoperative assessment techniques will be cost-effective compared to secondary surgery as both involve extra costs. This argument covers other described methods of intraoperative analysis of the SLNB, like touch imprint cytology and cytokeratin immunostain, as well [19].

Al together, when the benefit of avoiding reoperative axillary dissection is doubtful, routine intraoperative assessment by FS may be indicated only for a selected group of patients, for example, patients with larger tumours or higher age. Another option could be to perform SLNB in a separate procedure, perhaps under local anaesthesia. After pathologic results are known, definitive surgery for breast and axilla can be performed. However, nowadays, there is lively discussion about the need of treating the axilla in case of a positive SLN. The literature shows a very low axillary recurrence rate in case ALND is omitted after a positive SLN [20]. In this light, intraoperative FS probably will be less important in the future.

## 5. Conclusion

FS was associated with a higher FNR in our population, compared with the literature, and telepathology caused a decrease in sensitivity and a consequent increase in FNR. The benefits of this procedure, with this relative high FNR, are minimal. The SLN procedure itself is not time consuming, but since the benefit of avoiding reoperative axillary dissection is only 12.4%, routine FS may be indicated only for a selected group of patients, for example, patients with larger tumours. 

## Figures and Tables

**Table 1:** True and false results of FS by tumour size.

	T1	T2/T3
True negative FS (*n* = 447)	356	87/4
True positive FS (*n* = 78)	46	32
Total true results FS (*n* = 525, 83.6%)	**402**	**123**
False negative FS (*n* = 101)	65	35/1
False positive FS (*n* = 2)	—	2
Total false results FS (*n* = 103, 16.4%)	**65**	**38**
Sensitivity (%)	*41.4 *	*47.1 *

FS: frozen section.

**Table 2:** Relation between T-status, size of metastases, and sensitivity of the FS.

	Macro			Micro			ITC		
	*N*	FN	SE (%)	*N*	FN	SE (%)	*N*	FN	SE (%)
T1	58	22	62.0	36	28	22.2	17	16	5.9
T2/T3	43	13	69.8	16	13	18.9	8	8	—

Total	101	35	**65.3**	52	41	**21.1**	25	24	**4.0**

FS: frozen section, FN: false negative, SE: sensitivity.

**Table 3:** Relation between method of pathologic examination and sensitivity.

	*N*	Sensitivity (%)	FNR (%)
Pathologist in hospital	217	52.3	47.7
Telepathology, equipment 1	240	43.8	56.2
Telepathology, equipment 2	171	32.0	68.0

FNR: false negative rate.

**Table 4:** Results of frozen section analysis of sentinel lymph node biopsy in the literature.

Reference	Year	*N*	SNB+ (%)	SNB+/FS+	SNB+/FS−	FNR (%)
Wada [7]	2004	569	159 (28%)	133 (83.6%)	26	16.3%
Arora et al. [5]	2008	327	108 (33%)	78 (72.2%)	30	27.8%
McLaughlin et al. [8]	2008	931	306 (32.8%)	170 (55.6%)	136	44.4%
van de Vrande et al. [3]	2009	615	176 (28.6%)	126 (71.6%)	50	28.4%
Ali et al. [9]	2008	94	30 (33.3%)	23 (76.7%)	7	23.3%
Chan et al. [10]	2008	5298	1845 (34.8%)	1124 (60.9%)	721	39.1%
This study	—	628	179 (28.5%)	78 (43.6%)	101	56.4%

*N*: number of patients, SNB+: sentinel node biopsy with positive result, FS+: frozen section with positive result, FS−: frozen section with negative result, FNR: false negative rate.

## Abbreviations

ALND: Axillary lymph node dissection
Exc.: Excision
FN/FNR: False negative rate
FS: Frozen section
ITC: Isolated tumour cell
SE: Sensitivity
SN/SLN(B): Sentinel lymph node (biopsy)
WLE: Wide local excision.
